# Supplementary material for: Photoinduced loading of electron-rich Cu single atoms by moderate coordination for hydrogen evolution
Source: Nat Commun. 2022 Sep 20;13:5496. doi: 10.1038/s41467-022-33275-z (PMC9489781; doi:10.1038/s41467-022-33275-z)
Supplement: Supplementary file 1 — Supplementary Information [file 41467_2022_33275_MOESM1_ESM.pdf]

Supplementary Information

Photoinduced loading of electron-rich Cu single atoms by moderate coordination for hydrogen evolution

Weiwei Fu[1,a], Jin Wan[1,a], Huijuan Zhang[1,a], Jian Li[2], Weigen Chen[2], Yuke Li[3], Zaiping Guo[4], Yu Wang[1,2*]

[1] The School of Chemistry and Chemical Engineering, State Key Laboratory of Power Transmission Equipment & System Security and New Technology, Chongqing University, 174 Shazheng Street, Shapingba District, Chongqing City, 400044, PR China.

[2] The school of Electrical Engineering, Chongqing University, 174 Shazheng Street, Shapingba District, Chongqing City, 400044, China.

[3] Department of Chemistry, Centre for Scientific Modeling and Computation, Chinese University of Hong Kong, Shatin, Hong Kong 999077.

[4] School of Chemical Engineering and Advanced Materials, University of Adelaide, Adelaide, 5005, Australia.

[a] These authors contributed equally: Weiwei Fu, Jin Wan, Huijuan Zhang.

[*] E-mail: wangy@cqu.edu.cn.

Supplementary Notes

Supplementary Note 1. Chemicals. Pristine black phosphorus (99%) was purchased from Nanjing XFNANO Materials Tech Co. Cupric acetate monohydrate ($Cu(Ac)_2{\cdot}H_2O$), Cobalt(II) acetate tetrahydrate ($Co(Ac)_2{\cdot}4H_2O$), and *N*-methyl-2-pyrrolidone (NMP) were obtained from Sigma Aldrich. All chemical reagents were used without further purification.

Supplementary Note 2. Characterizations. TEM and EDS characterizations were achieved using three TEM instruments (JEOL JEM-2100, FEI Talos F200X and FEI Titan Cubed Themis G2 300 with a probe corrector). High-angle annular dark-field STEM energy dispersive X-ray spectroscopy (HAADF-STEM-EDS) were conducted on a JEOL GrandARM300F scanning transmission electron microscope with double Cs correctors at an acceleration voltage of 300 kV. X-ray photo electron spectrometer (XPS) with an ESCALAB 250Xi from Thermo scientific using monochromatic Al Kα radiation (225W, 15mA, 15kV), powder X-ray diffraction (XRD, X'Pert Powder with Cu Kα radiation), Raman spectrometer equipped with argon (532 nm) laser in the wavenumber of 100-2000 cm-1 (Horiba LabRAM HR Evolution) were adopted to characterize the as-prepared samples. The metal loadings were tested by ICP-MS on a VISTA MPX (Varian, Inc.). EPR measurements were implemented at 5.7 K by a Bruker A300 spectrometer, which operated in the X-band (9.64 GHz) and 100 kHz magnetic field modulation with 10 G modulation amplitude.

Supplementary Note 3. The turnover frequency (TOF). To reveal the activity of each Cu and Co active site, the TOFs of n-Cu/BP, n-Co/BP and CoCu/BP catalysts are calculated, respectively. Per copper and cobalt site of was calculated according to the following equation:

$$TOF = \frac{number\ of\ total\ hydrogen\ turnovers/cm^2\ of\ geometric\ area}{number\ of\ active\ sites/cm^2\ of\ geometric\ area} \quad (1)$$

The total number of hydrogen turnovers was calculated from the current density according to:

$$no.of\ H_2 = (J\ mA\ cm^{-2})\left(\frac{1C\ s^{-1}}{1000\ mA}\right)\left(\frac{1mol\ of\ e^-}{96485.3\ C}\right)\left(\frac{1\ mol\ of\ H_2}{2mol\ of\ e^-}\right)\left(\frac{6.022\times10^{23}\ H_2\ moleculars}{1mol\ H_2}\right)$$

$$= J \times 3.12 \times 10^{15}\ H_2 s^{-1} cm^{-2}\ per\ mA\ cm^{-2} \quad (2)$$

The Cu and Co content of n-Cu/BP and n-Co/BP determined by the ICP-OES is ca. 11.3 and 5.2 wt%, respectively, and that of in CoCu/BP is 8.8 and 3.7 wt%, respectively. The mass loading on the electrode is ~0.57 mg cm^{-2}. Thus, n is calculated as:

$$N_{active}^{n-\frac{Cu}{BP}} = \left(\frac{Cu\ wt\%\times catalyst\ loading\ per\ geometric\ area\ (g\ cm^{-2})}{Cu\ Mw\ (g\ mol^{-1})}\right)\times\left(\frac{6.022\times10^{23}}{1\ mol}\right)$$

$$= \frac{11.3\%\times0.57\ mg\ cm^{-2}}{(63.55)\ g\ mol^{-1}}\times\left(\frac{6.022\times10^{23}}{1\ mol}\right) = 6.10\times10^{17}\ mol\ cm^{-2} \quad (3)$$

$$N_{active}^{n-\frac{Co}{BP}} = \left(\frac{Co\ wt\%\times catalyst\ loading\ per\ geometric\ area\ (g\ cm^{-2})}{Co\ Mw\ (g\ mol^{-1})}\right)\times\left(\frac{6.022\times10^{23}}{1\ mol}\right)$$

$$= \frac{5.2\%\times0.57\ mg\ cm^{-2}}{(58.93)\ g\ mol^{-1}}\times\left(\frac{6.022\times10^{23}}{1\ mol}\right) = 3.03\times10^{17}\ mol\ cm^{-2} \quad (4)$$

$$N_{active}^{\frac{CuCo}{BP}} = \left(\frac{CuCo\ wt\%\times catalyst\ loading\ per\ geometric\ area\ (g\ cm^{-2})}{{}^2/_3 Cu\,{}^1/_3 Co\ Mw\ (g\ mol^{-1})}\right)\times\left(\frac{6.022\times10^{23}}{1\ mol}\right)$$

$$= \frac{12.5\%\times0.57\ mg\ cm^{-2}}{(63.55\times{}^2/_3+58.93\times{}^1/_3)\ g\ mol^{-1}}\times\left(\frac{6.022\times10^{23}}{1\ mol\ C}\right) = 6.92\times10^{17}\ mol\ cm^{-2} \quad (5)$$

Finally, the current density from the LSV polarization curve can be converted into TOF values according to:

$$TOF_{n-Cu/BP} = 0.0051 \times |J| \tag{6}$$

$$TOF_{n-Co/BP} = 0.0103 \times |J| \tag{7}$$

$$TOF_{CuCo/BP} = \frac{|J| \times 3.12 \times 10^{15} H_2 s^{-1} cm^{-2}\ per\ mA\ cm^{-2}}{N_{active}^{CuCo/BP}} = 0.0045 \times |J| \tag{8}$$

Supplementary Figures

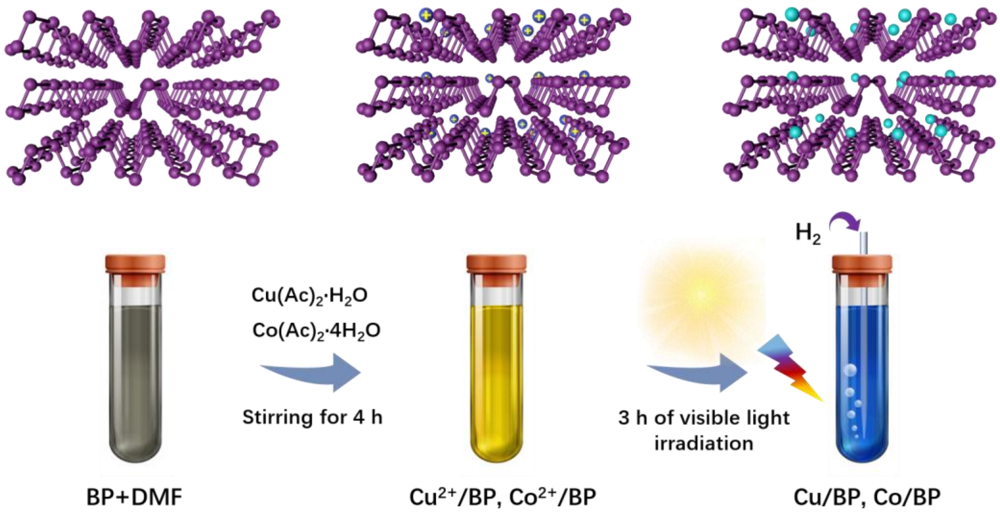

Supplementary Figure 1. Schematic illustration the photochemical process.

The visible light-induced formation of SACs on BP is shown (the atomic structure of BP and tubes were drawn by Cinema 4D software).

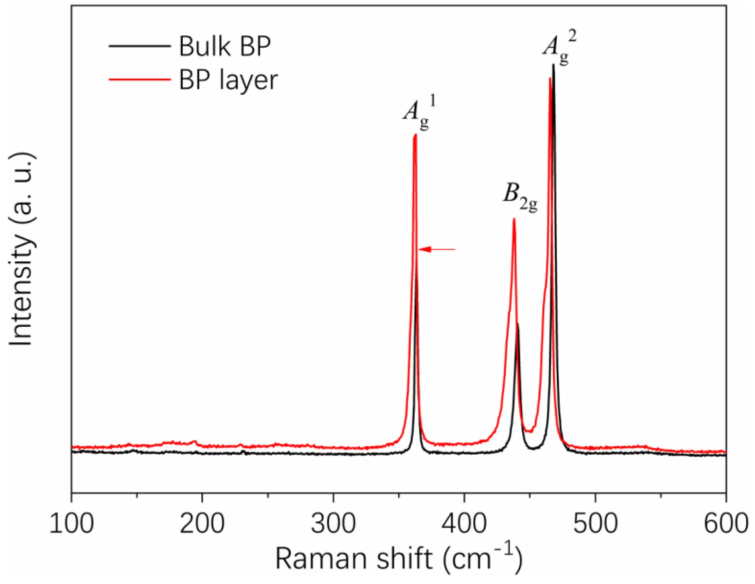

Supplementary Figure 2. Raman characterizations of bulk BP and BP layer.

Raman shifts of bulk BP and exfoliated BP layer. Raman spectra of bulk BP and exfoliated BP sheets show three typical peaks between 300 and 500 cm^{-1}, which include an out-of-plane vibrational mode of A_g^1, two in-plane modes of B_{2g} and A_g^2[1]. Compared to bulk BP, the Raman signals of exfoliated BP are shifted to a high number, which is in agreement with previous reports[2].

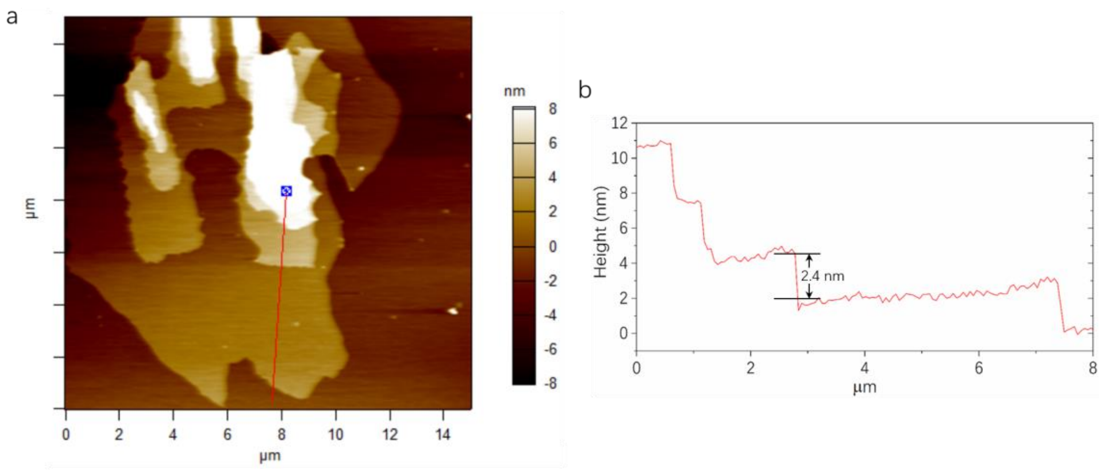

Supplementary Figure 3. AFM characterizations of BP layer.

AFM image of exfoliated BP layer.

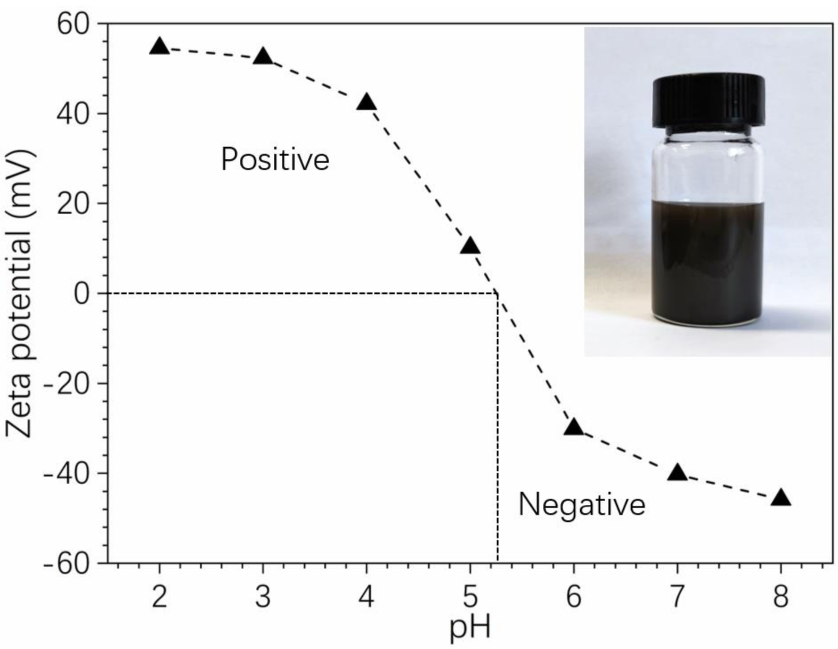

Supplementary Figure 4. Zeta-potential characterizations of BP nanosheets.

The zeta-potential of BP nanosheets dispersed in NMP of different pH values, showing a negative surface potential in a neutral environment. Cu^{2+} and Co^{2+} ions are readily adsorbed onto the negative surface of BP by electrostatic interaction. In addition, the value of zeta potential in BP surface is above 40 mV in a neutral environment, indicating that BP nanosheets possess good dispersion in NMP, as shown in the illustration.

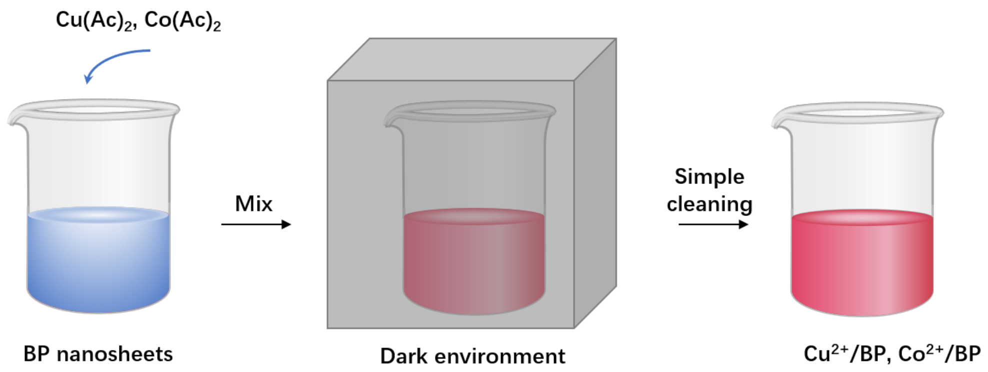

Supplementary Figure 5. Scheme for the synthesis of Cu^{2+}/BP and Co^{2+}/BP

Cu^{2+}/BP and Co^{2+}/BP were synthesized without vis irradiation and H_2 auxiliary.

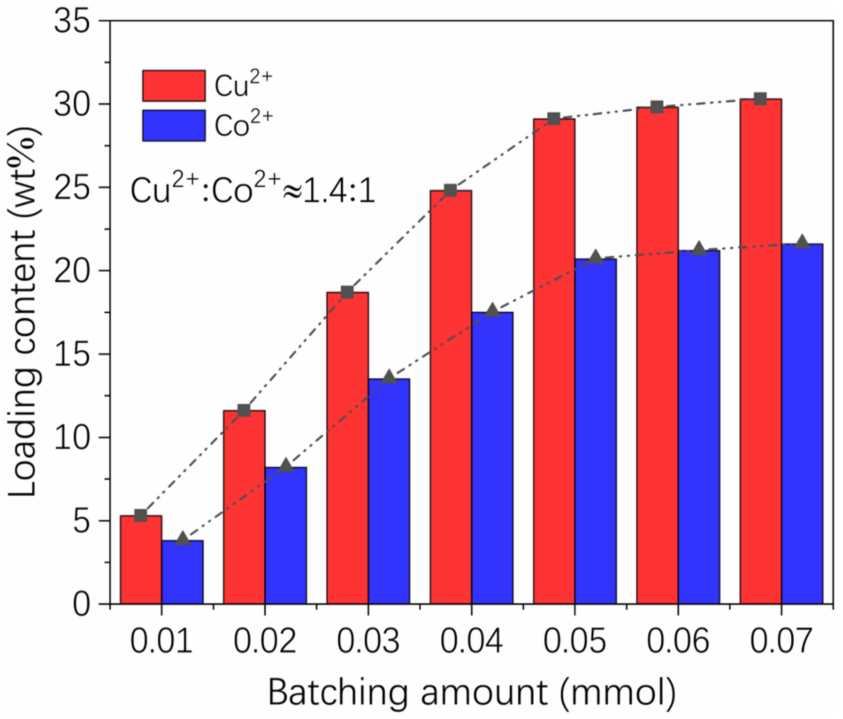

Supplementary Figure 6. ICP characterizations.

The loading contents of Cu^{2+} and Co^{2+} for BP nanosheets in various acetate solution (the molar ratio of $Cu(Ac)_2{\cdot}H_2O$ to $Co(Ac)_2{\cdot}4H_2O$ was 1:1) containing different concentrations of Cu or Co. The loading concentrations of Cu^{2+} and Co^{2+} are expressed as the weight percentage related to support. The loading amount of was evaluated by ICP-OES analysis. The results indicate that metal ions of Cu and Co show high adsorption capacity in BP and gradually tend to be saturated. The mass percentage of Cu^{2+} and Co^{2+} is close to 1.4:1 in Cu^{2+}/BP and Co^{2+}/BP.

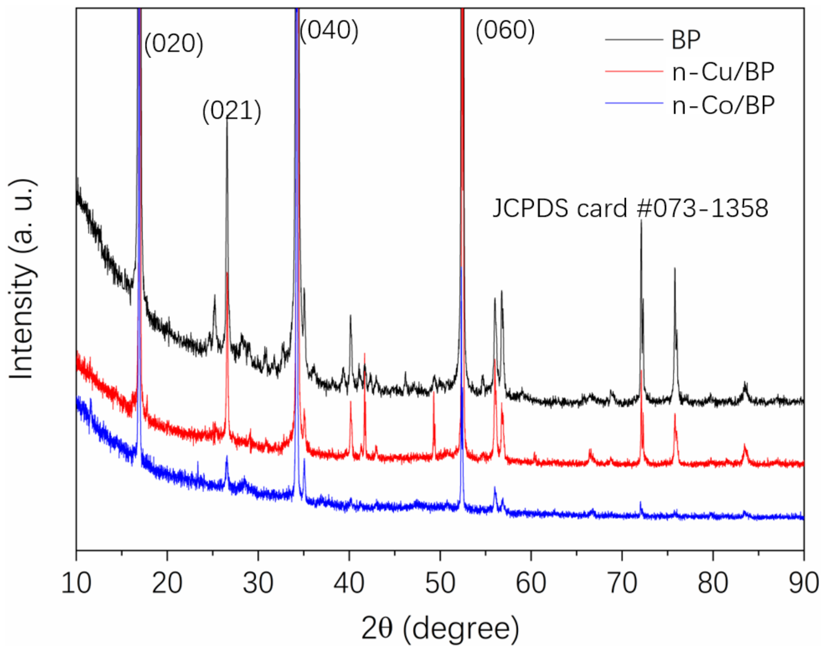

Supplementary Figure 7. XRD characterizations.

The X-ray diffraction (XRD) of BP, n-Cu/BP and n-Co/BP nanosheets.

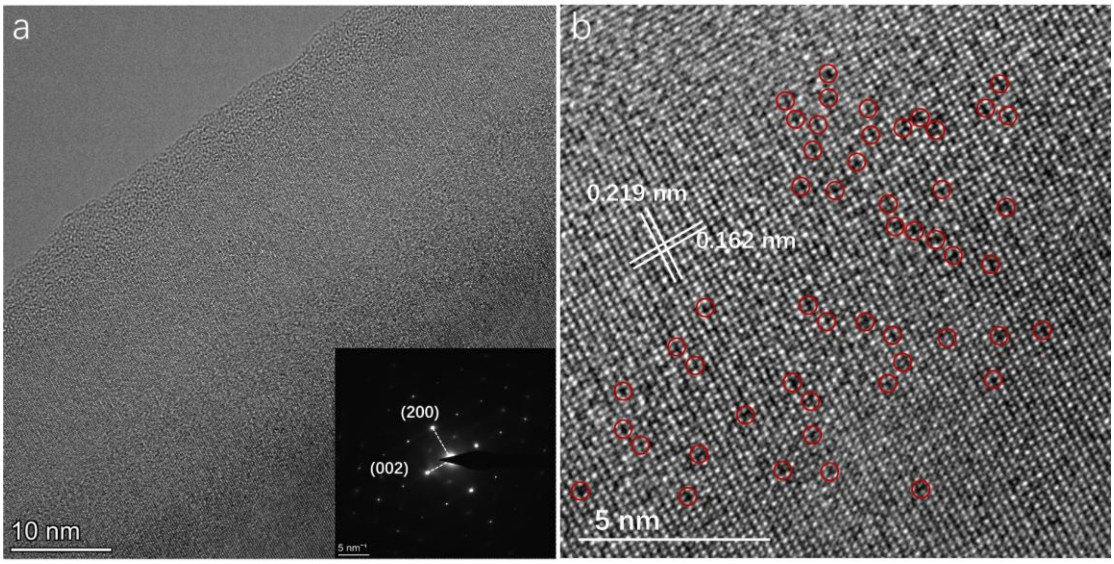

Supplementary Figure 8. STEM characterizations.

A STEM images of M/BP and the corresponding SAED analysis indicate the well-crystal structure of BP. The samples go through a “organic impurity removal” step before testing STEM to obtain stable signals.

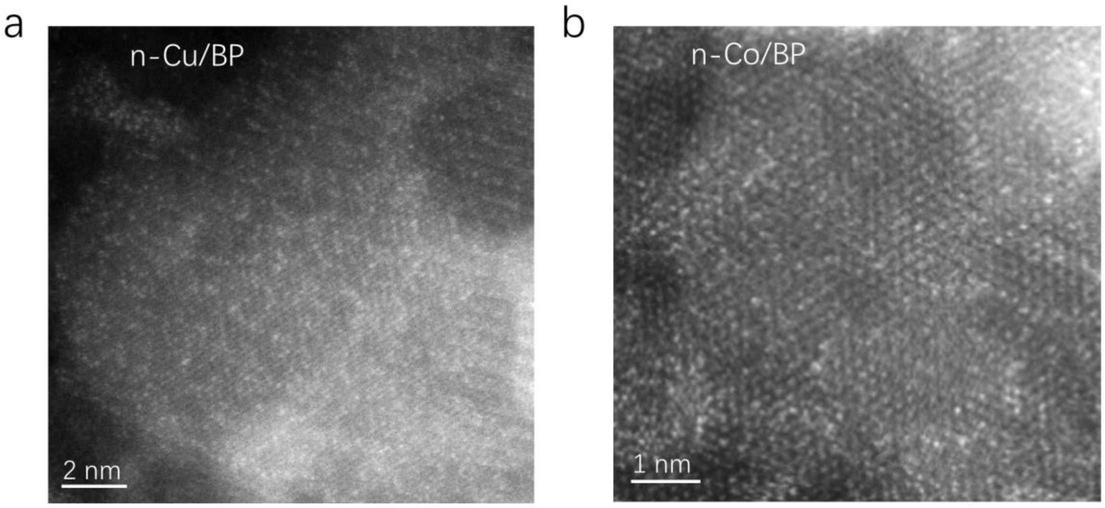

Supplementary Figure 9. HAADF-STEM characterizations of n-Cu/BP and n-Co/BP.
HAADF-STEM image of n-Cu/BP (a) and n-Co/BP (b).

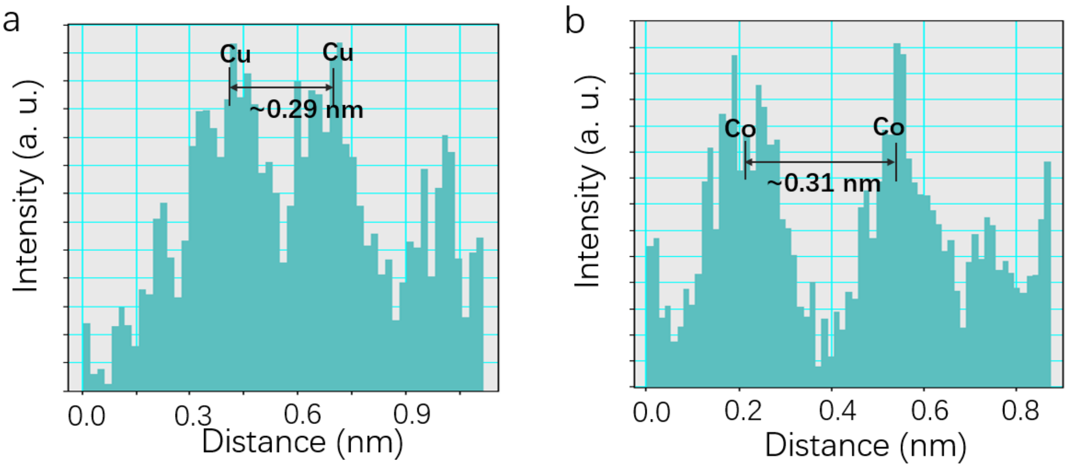

Supplementary Figure 10. Their intensity profile analyses by HAADF-STEM.
The intensity profiles along the line X–Y in n-Cu/BP and n-Co/BP.

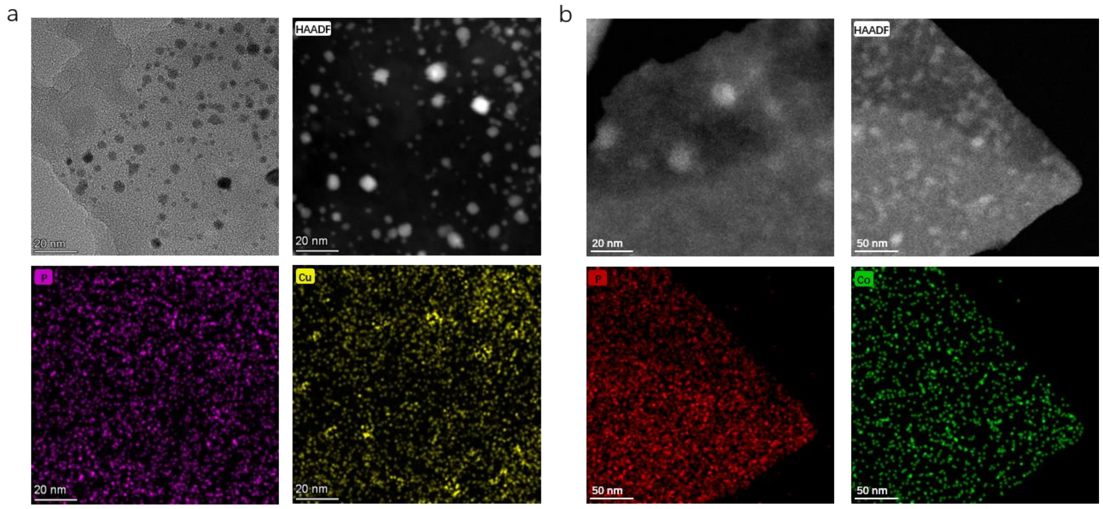

Supplementary Figure 11. TEM-EDX element mapping.

Representative TEM images and EDS mapping of supported Cu and Co nanoparticles (Cu NPs/BP and Co NPs/BP) prepared by a conventional impregnation method followed by conventional thermal reduction in 500 °C. The formation of Cu and Co nanoparticles was clearly observed in the catalyst.

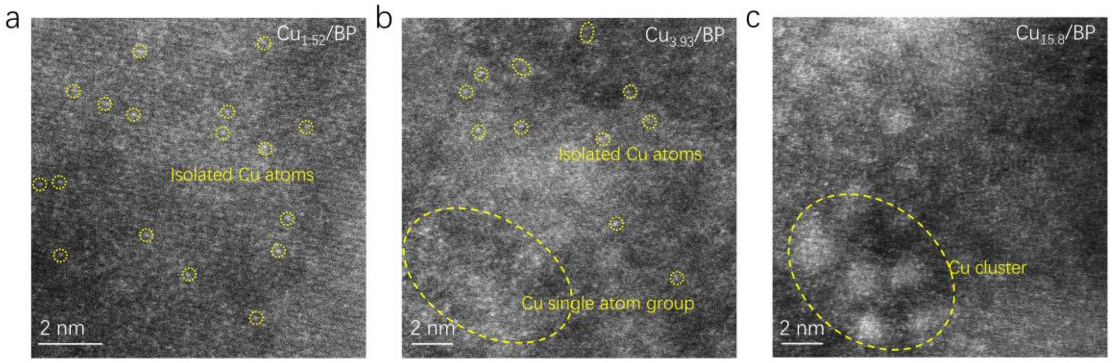

Supplementary Figure 12. HAADF-STEM characterizations.

HAADF-STEM images of $Cu_{1.52}$/BP, $Cu_{3.92}$/BP, and $Cu_{15.8}$/BP. These samples were synthesized under different vis irradiation time with H_2 auxiliary for 0.5, 1, and 5 h, respectively.

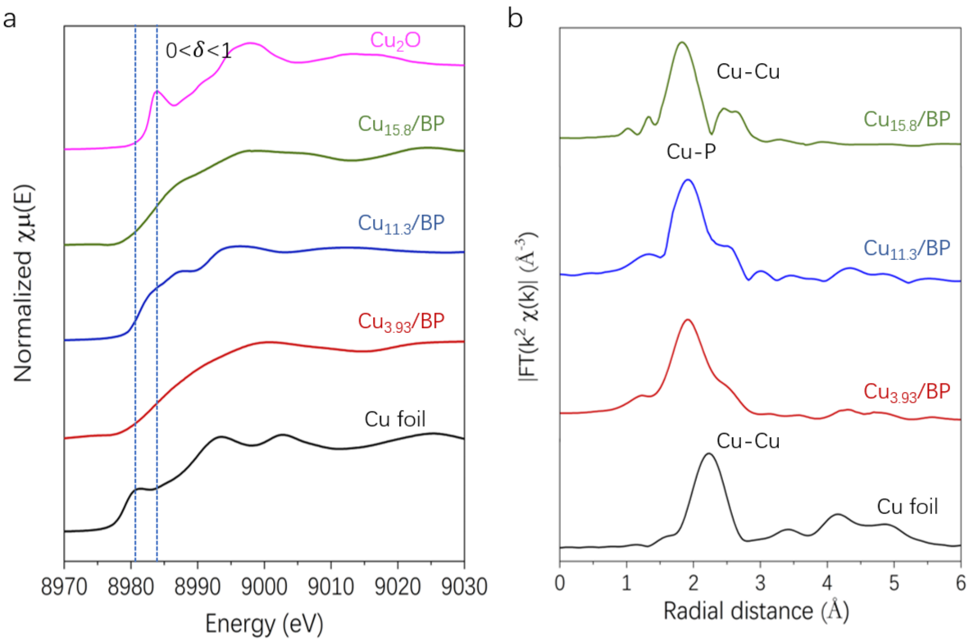

Supplementary Figure 13. XANES and FT k^2-weighted EXAFS spectra.

a Cu k-edge XANES. **b** FT k^2-weighted EXAFS spectra of Cu/BP and the reference Cu foil and Cu_2O.

The Cu K edge XANES spectra of Cu foil and Cu/BP with different Cu loading are shown in **Supplementary Fig. 13 a**. The near-edge features of Cu/BP are in between of those of Cu foil and Cu_2O, indicating that the Cu species are partially positively charged ($Cu^{\delta+}$, $0<\delta<1$) due to the charge redistribution between Cu^0 and BP. Fourier-transformed k^2-weighted EXAFS in R space shows that $Cu_{3.93}$/BP and $Cu_{11.3}$/BP possess one main peak at 1.92 Å from the first coordination shell of Cu-P bond (**Supplementary Fig. 13 b**). $Cu_{15.8}$/BP displays an additional minor peak at 2.4 Å, ascribed to Cu-Cu scattering, confirming the formation of Cu clusters. By contrast, no obvious peaks at 2.4 Å for $Cu_{11.3}$/BP and $Cu_{3.93}$/BP evidence that Cu atoms are atomically dispersed, in accordance with the HAADF-STEM observations (**Supplementary Fig. 12**).

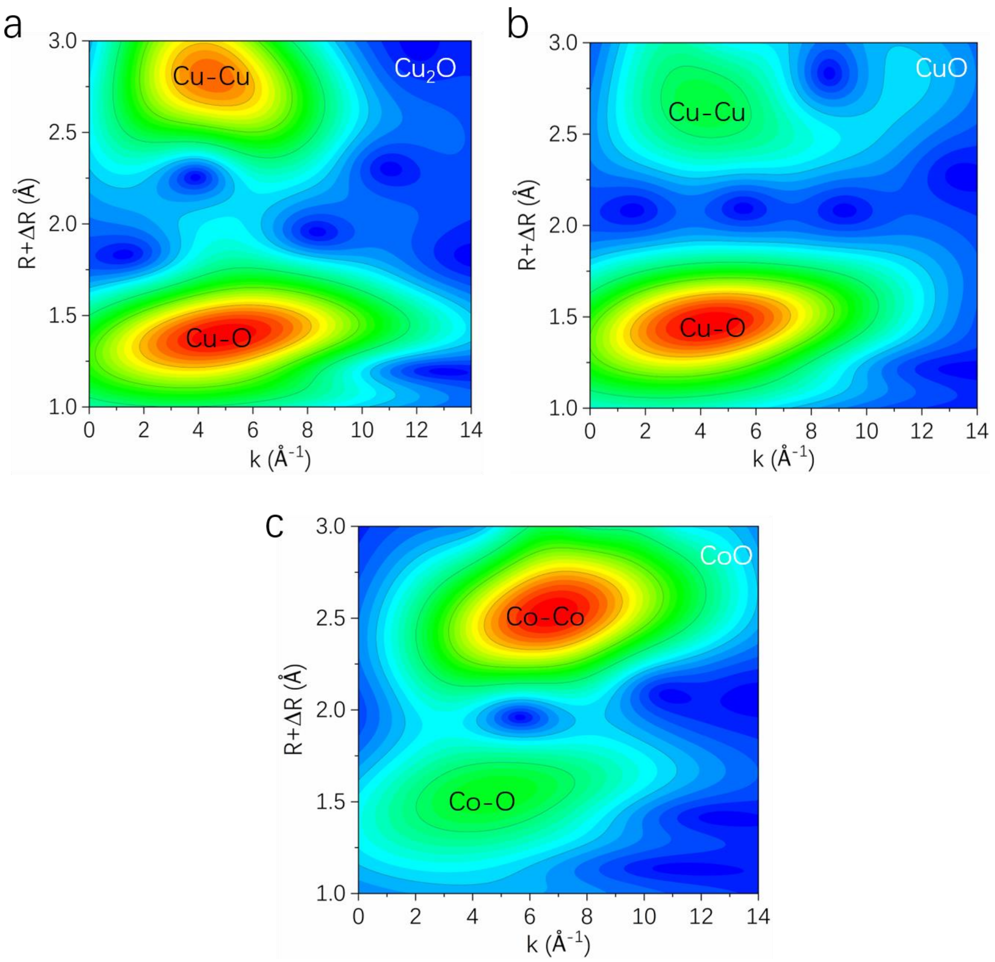

Supplementary Figure 14. The wavelet-transform images.

a,b WT contour plots of Cu K-edge at R space for Cu_2O and CuO. **c** Co K-edge at R space for CoO.

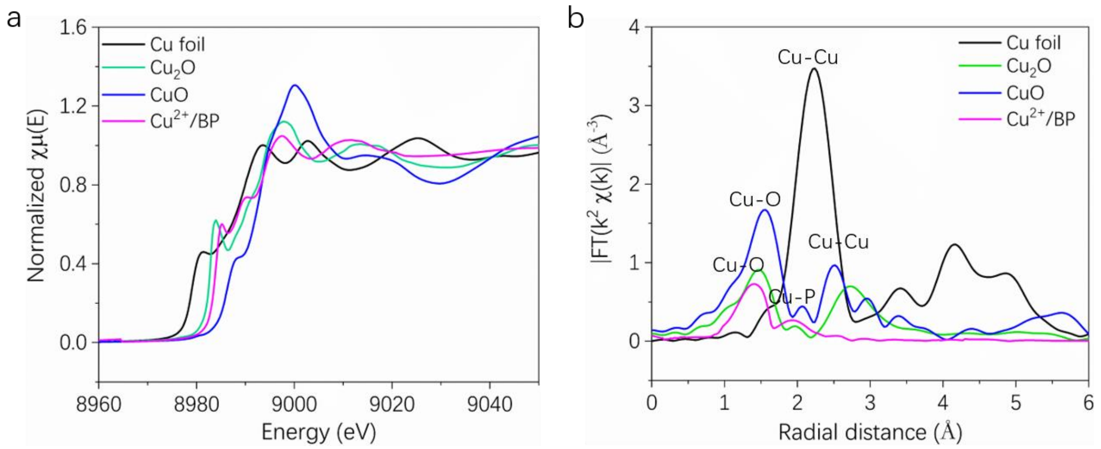

Supplementary Figure 15. XANES and FT k^2-weighted EXAFS spectra of Cu^{2+}/BP.

a The normalized Cu K-edge XANES and **b** FT-EXAFS spectra of Cu^{2+}/BP, Cu foil, Cu_2O and CuO.

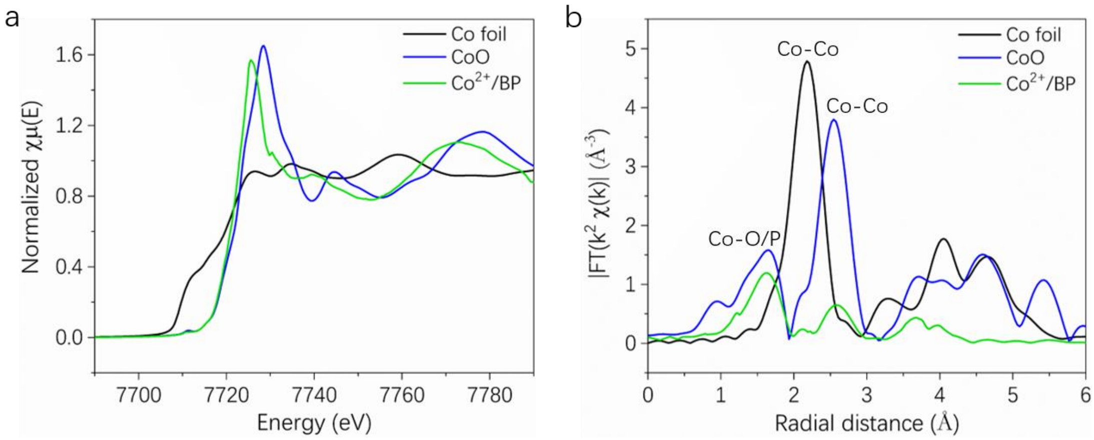

Supplementary Figure 16. XANES and FT k^2-weighted EXAFS spectra of Co^{2+}/BP.

a The normalized Co K-edge XANES and **b** FT-EXAFS spectra of Co^{2+}/BP, Co foil and CoO.

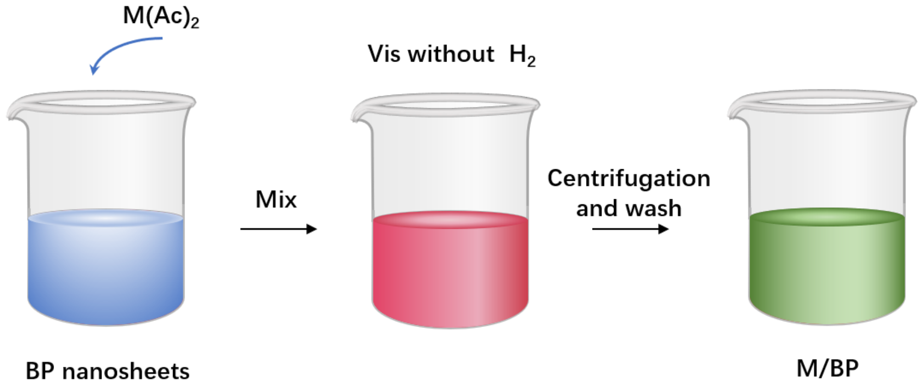

Supplementary Figure 17 Scheme for the synthesis of M/BP under vis irradiation without H_2 auxiliary.

After $Cu(Ac)_2$ (0.05 mmol) or $Co(AC)_2$ (0.05 mmol) was introduced into NMP dispersion of BP nanosheets to allow the adsorption of M^{2+} species, and then the mixture was treated with vis light without H_2 auxiliary for 3h. The sample was thoroughly washed by ethanol and water.

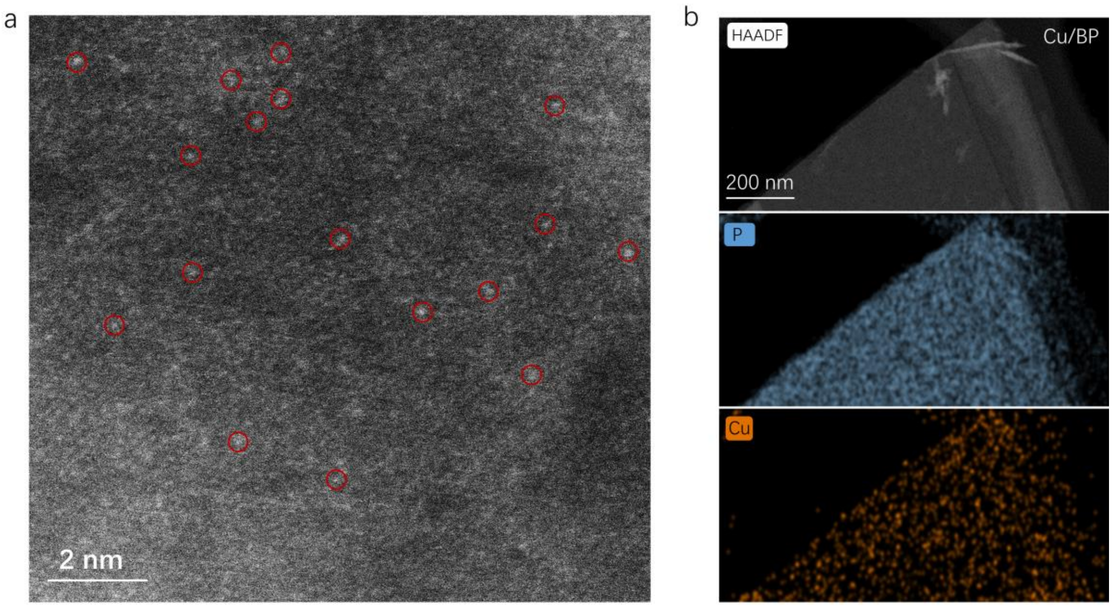

Supplementary Figure 18. HAADF-STEM characterizations and STEM-EDX element mapping.

a Aberration-corrected scanning transmission electron microscopy (STEM) images of Cu/BP prepared under vis irradiation without H_2 auxiliary. **b** STEM-EDS elemental mapping of corresponding Cu/BP nanosheet.

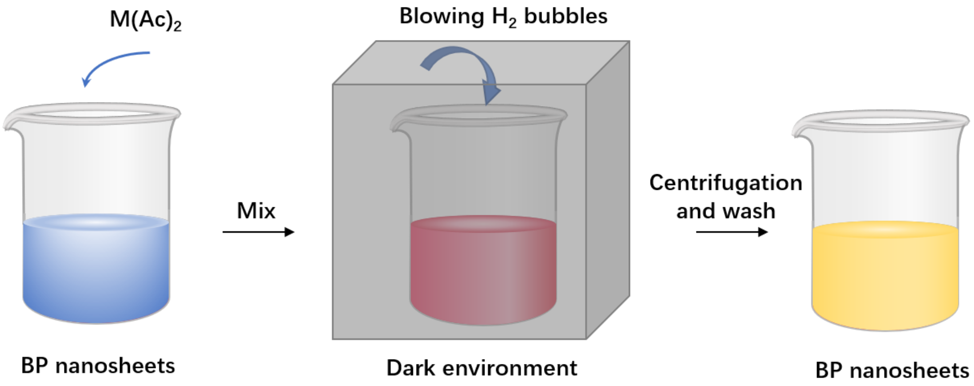

Supplementary Figure 19. Scheme for the H_2 treatment for BP nanosheets.

The BP nanosheets in NMP with H_2 treatment under dark environment for 3h, and then the sample was thoroughly washed by ethanol and water.

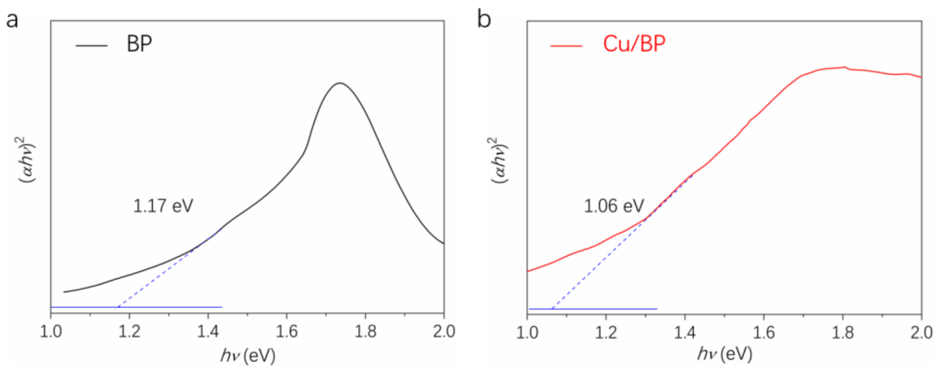

Supplementary Figure 20. UV/Vis diffuse reflectance spectra.

Plots of $(\alpha h\nu)^2$ versus the energy of the exciting light ($h\nu$) curve of BP and Cu/BP based on Tauc plot equation: $[(\alpha h\nu)^2 = A(h\nu\text{-}E_g)]$. BP is direct bandgap materials, thus r=2 for Tauc plot $(\alpha h\nu)^r$ versus $h\nu^3$.

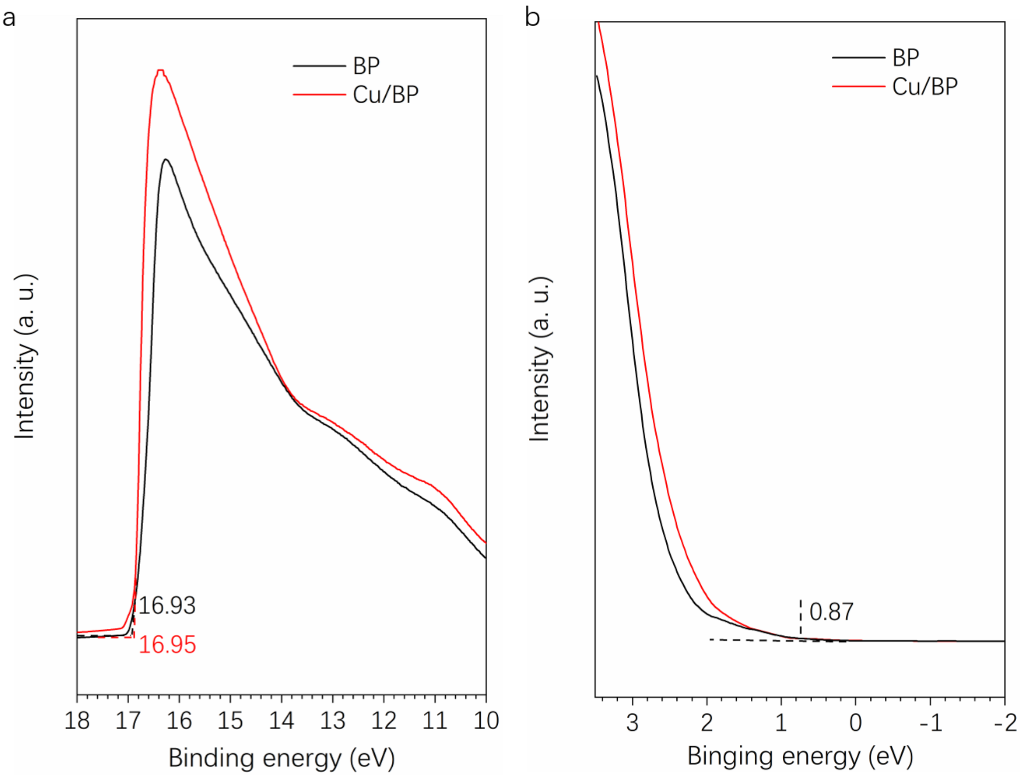

Supplementary Figure 21. The ultraviolet photoelectron spectra.

UPS spectra of BP and Cu/BP. We used ultraviolet photoelectron spectroscopy (UPS) to determine the ionization potential, which is equivalent to the valence band energy (E_{VB}). According to the linear intersection method, the E_{VB} of BP and Cu/BP were calculated to be 5.16 and 5.14 eV (vs vacuum), respectively, by subtracting the width of the He I UPS spectrum from the excitation energy (21.22 eV). The E_{VB} values in eV can be converted to the electrochemical energy potential of 0.72 and 0.7 eV, respectively, according to the reference standard for which 0 V vs. RHE equals -4.44 eV vs. evac (vacuum level).

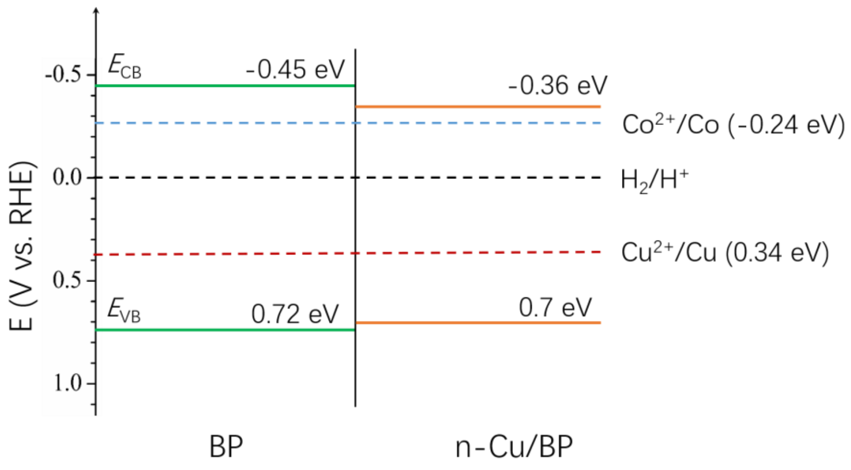

Supplementary Figure 22. The energy level diagrams of BP and Cu/BP.

Schematic illustration of the energy level information from UPS, in which E_{CB} is conduction band energy, and E_{VB} is valence band energy.

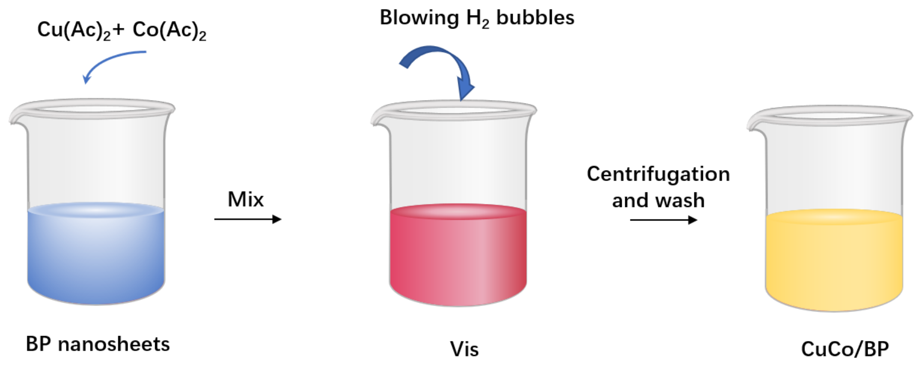

Supplementary Figure 23. Scheme for the synthesis of bimetallic CuCo/BP.

After $Cu(Ac)_2$ and $Co(Ac)_2$ (both 0.05 mmol) were introduced into NMP dispersion of BP nanosheets to allow the adsorption of M^{2+} species, and then the mixture was treated with vis light with H_2 auxiliary for 3h. The sample was thoroughly washed by ethanol and water.

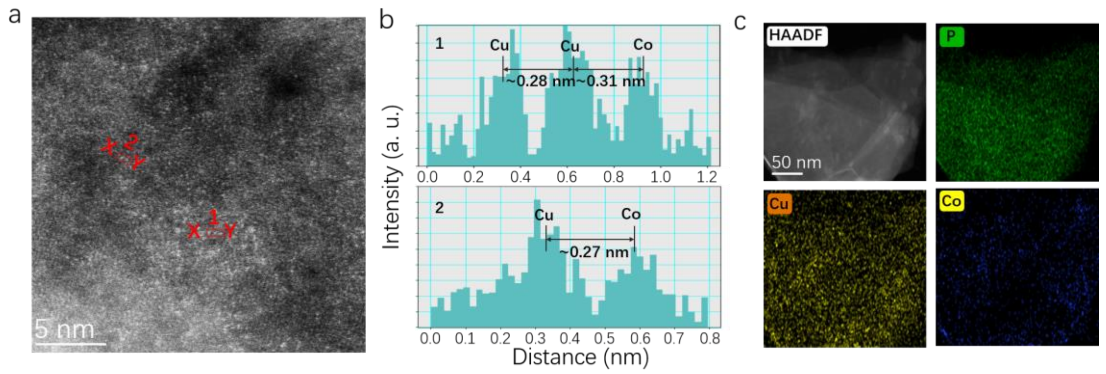

Supplementary Figure 24. HAADF-STEM characterization and STEM-EDS elemental mapping.

a HAADF-STEM image of CuCo/BP. **b** The corresponding intensity profiles along the line X–Y in (a). **c** STEM-EDS elemental mapping of CuCo/BP nanosheet.

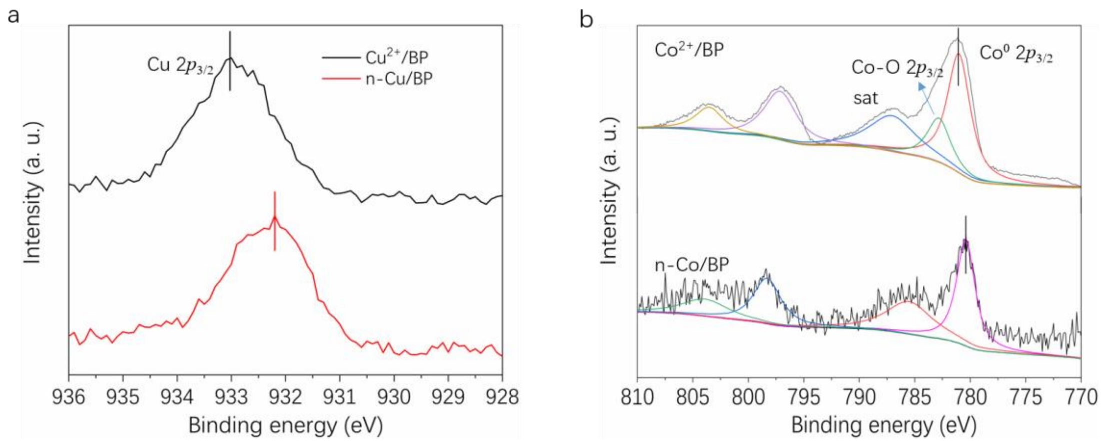

Supplementary Figure 25. XPS characterization.

a Cu 2*p* XPS spectra of Cu^{2+}/BP and n-Cu/BP catalysts. **b** Co 2*p* XPS spectra of Co^{2+}/BP and n-Co/BP catalysts.

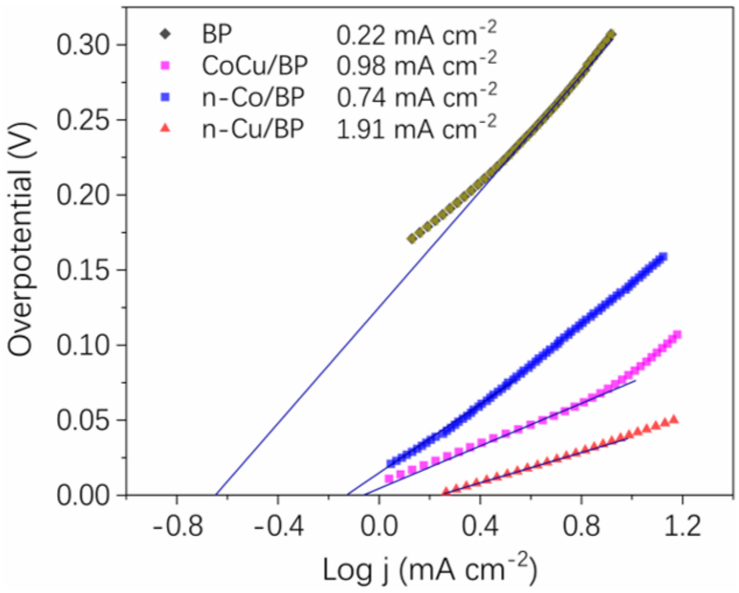

Supplementary Figure 26. The analysis of exchange current densities.
The exchange current densities of the n-Cu/BP, n-Co/BP, CoCu/BP, and BP electrocatalysts.

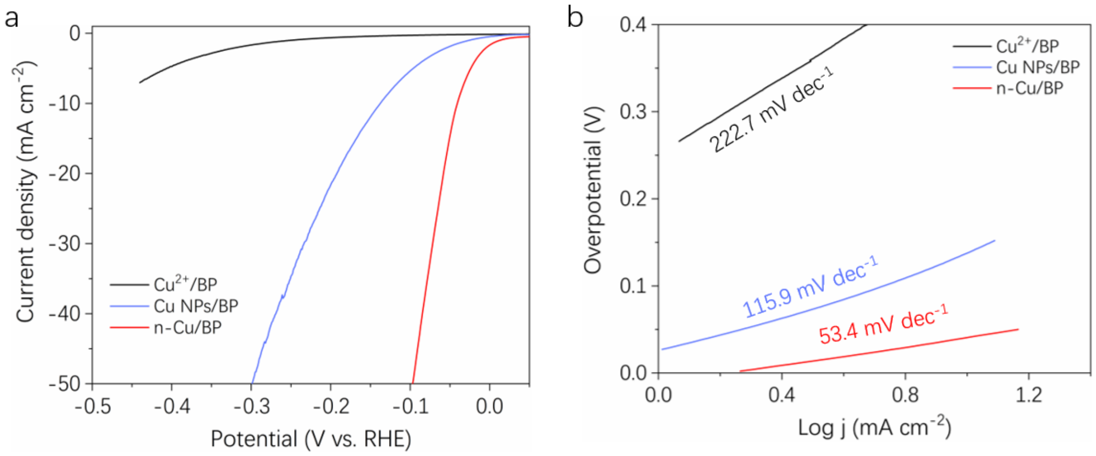

Supplementary Figure 27. LSV and Tafel slope curves.
HER performances of Cu^{2+}/BP, Cu NPs/BP, and n-Cu/BP.

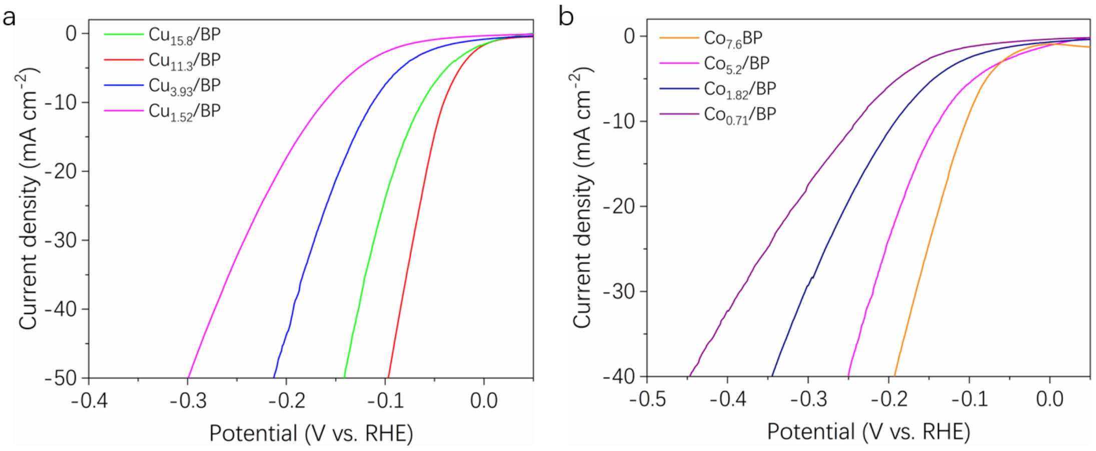

Supplementary Figure 28. LSV curves of Cu/BP and Co/BP.

HER performances of Cu/BP and Co/BP under different vis irradiation time (0.5, 1, 3, and 5h) with H_2 auxiliary, respectively.

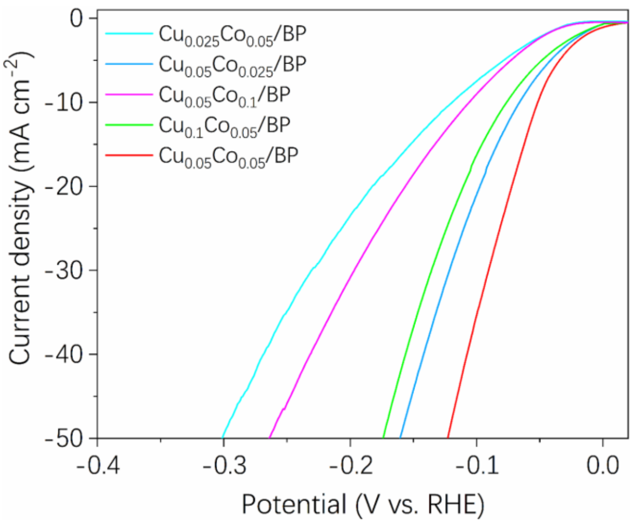

Supplementary Figure 29. LSV curves of CuCo/BP.

HER performances of CuCo/BP with different Cu and Co feeding amount.

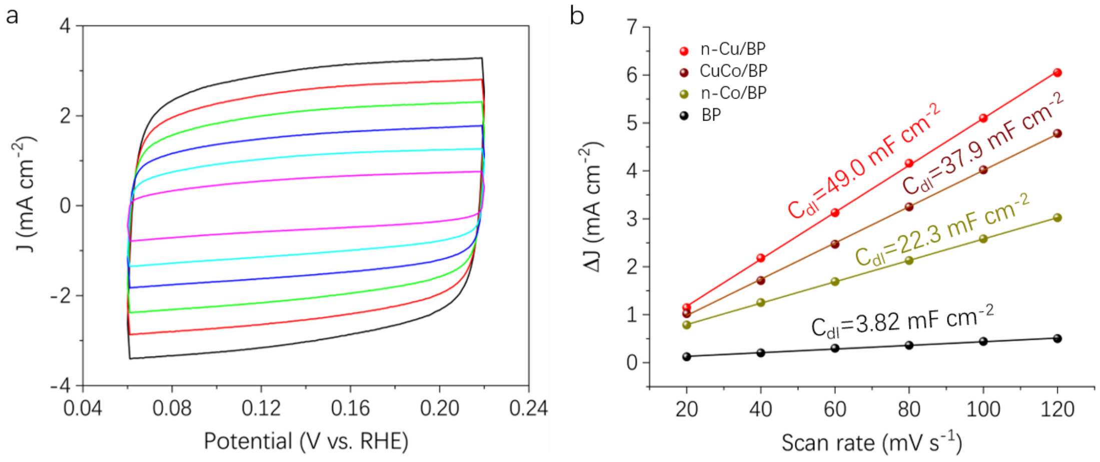

Supplementary Figure 30. The double-layer capacitance analysis.

a Typical cyclic voltammetry curves (CVs) of n-Cu/BP with different scan rates at 20, 40, 60, 80, 100, and 120 in the potential range of 0.06-0.22 V, respectively. **b** Capacitive current at 0.14 V based on scan rate for n-Cu/BP, n-Co/BP, CuCo/BP, and BP ($\Delta J_0=(J_a-J_c)$, J respects current density).

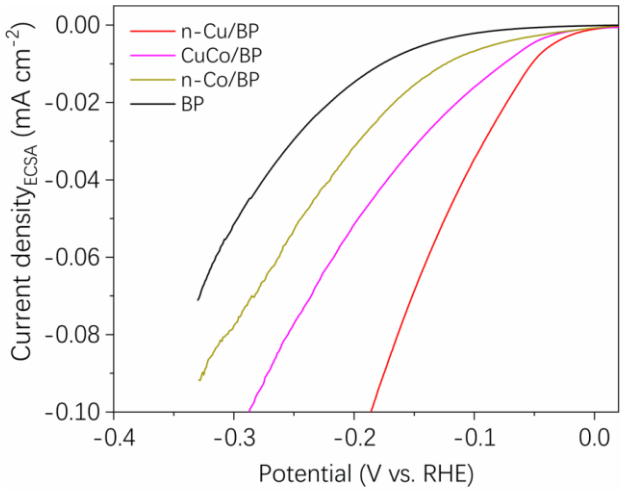

Supplementary Figure 31. ECSA normalized HER polarization curves.

ECSA normalized HER polarization curves of the n-Cu/BP, CuCo/BP, n-Co/BP, and BP electrodes.

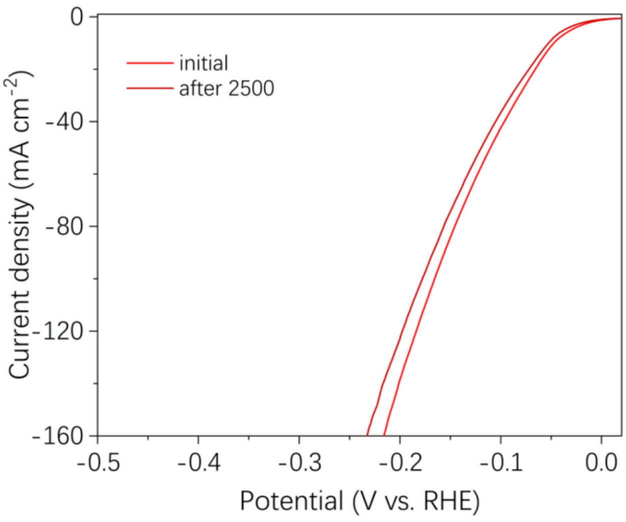

Supplementary Figure 32. CV stability test.

CV stability of the CuCo/BP catalyst.

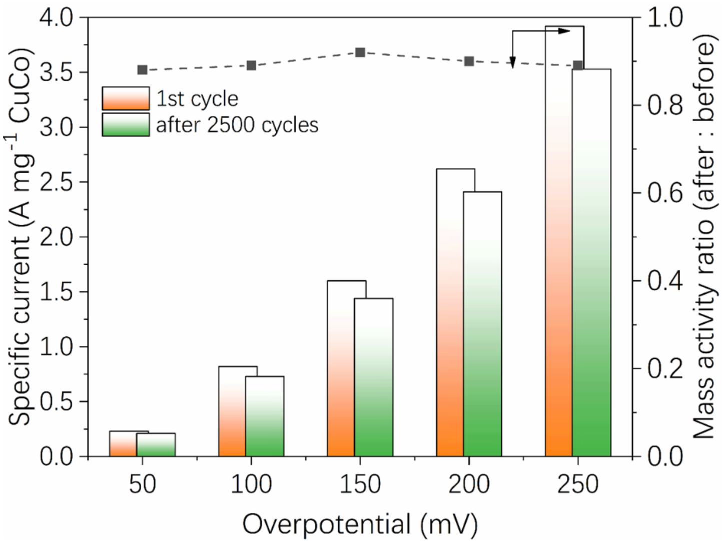

Supplementary Figure 33. Specific current activities of n-Cu/BP at different overpotential.

Specific current of n-Cu/BP with 1st and after 2500 cycles of CV, and corresponding mass activity ratio between before and after 2500 CV cycles at different overpotential.

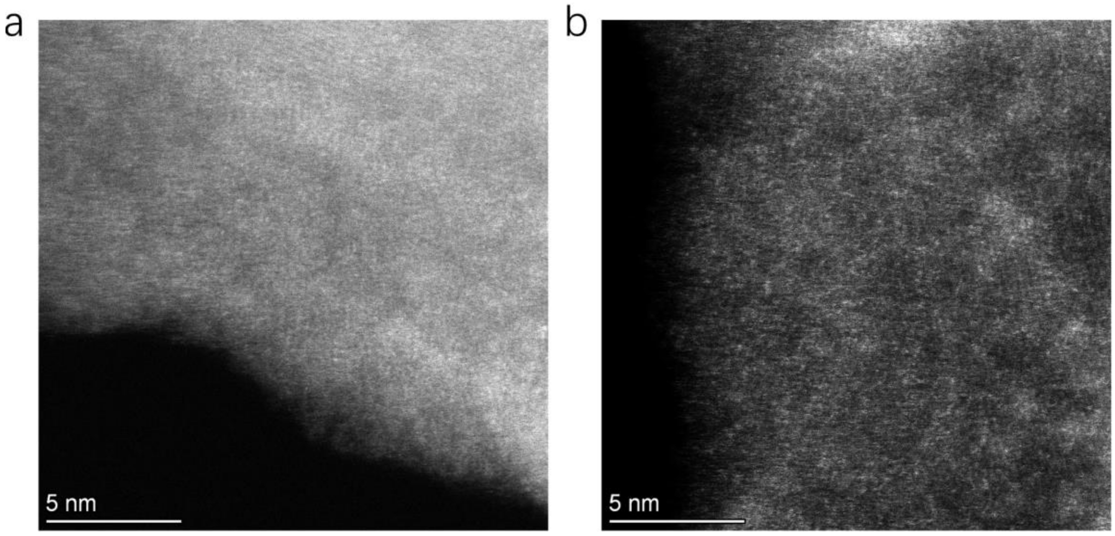

Supplementary Figure 34. HAADF-STEM characterization.
The image of n-Cu/BP by aberration-corrected transmission electron microscope after long-term electrocatalysis.

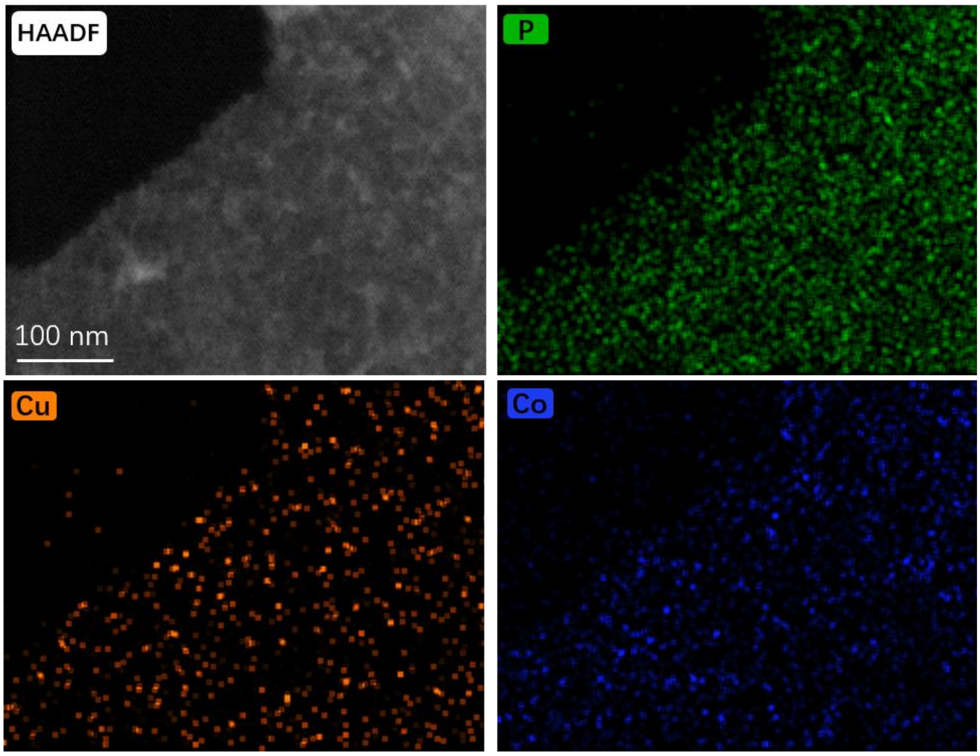

Supplementary Figure 35. HAADF-STEM image and element mapping characterization.
HAADF-STEM image and element mapping of n-Cu/BP after long-term electrocatalysis.

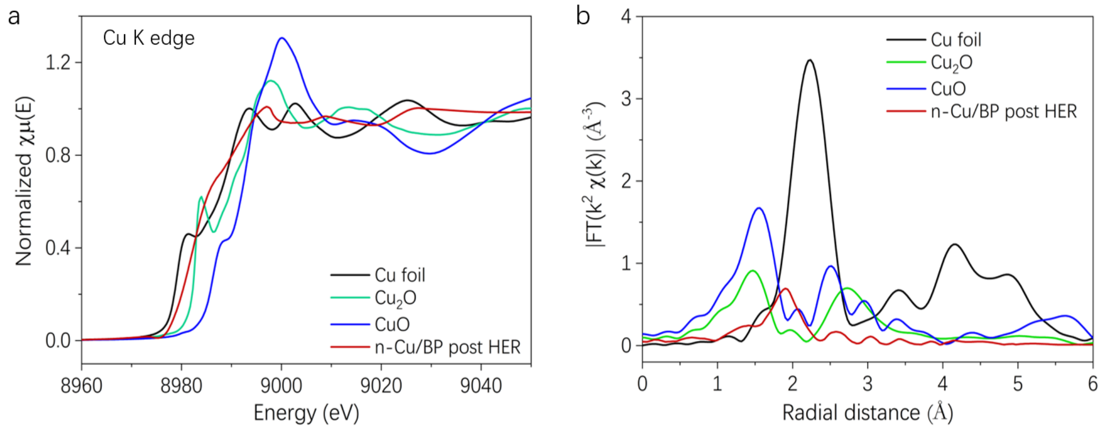

Supplementary Figure 36. XANES and FT k^2-weighted EXAFS spectra.

XAS characterizations after long-time operation. **a** XANES and **b** corresponding FT-EXAFS spectra for n-Cu/BP post HER, Cu foil, Cu_2O, and CuO.

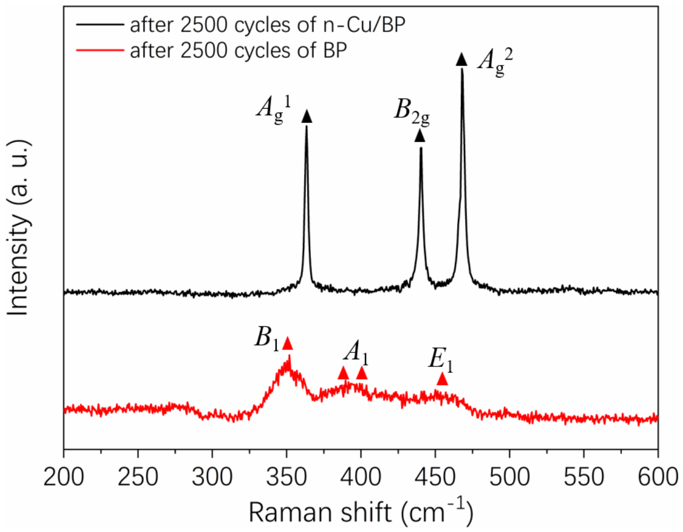

Supplementary Figure 37. Raman characterization.

Raman shifts of n-Cu/BP and BP nanosheets after 2500 CV cycles.

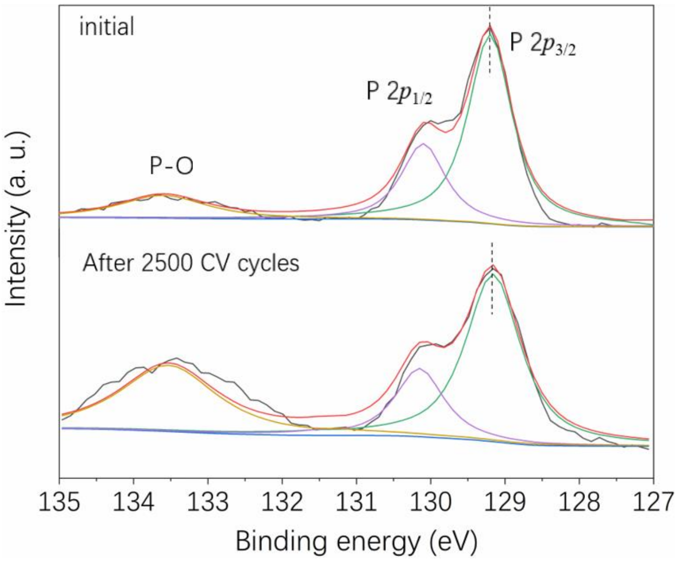

Supplementary Figure 38. XPS characterization for P 2*p* XPS spectra.

P 2*p* XPS spectra of n-Cu/BP after long-term CV cycles for HER. There is no obvious shift for the P 2*p* XPS for n-Cu/BP. The intensity of P-O bonds in n-Cu/BP is higher after long-term CV cycles, which is ascribed to its low surface absorbability to OH^- or PO_x.

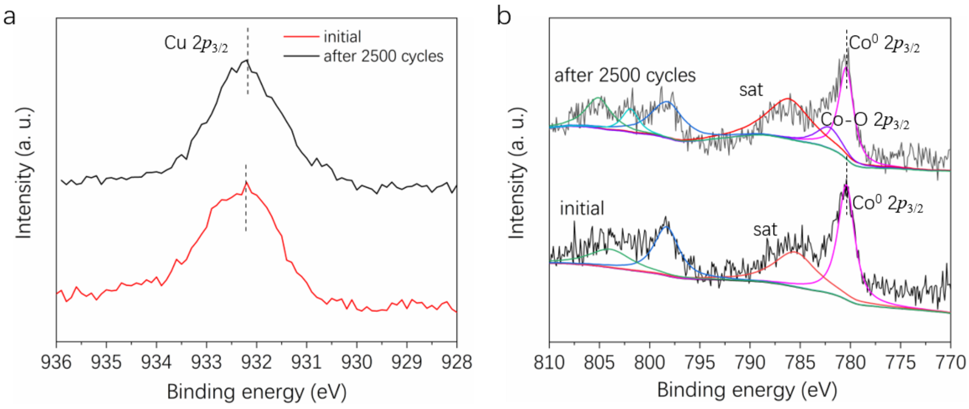

Supplementary Figure 39. XPS characterization for Cu 2*p* and Co 2*p* XPS spectra.

a Cu 2*p* and (**b**) Co 2*p* XPS spectra of n-Cu/BP after long-term CV cycles for HER. There is a little change for Cu 2*p* and Co 2*p* XPS spectra after long-term CV cycles, indicating the excellent structural stability of n-Cu/BP. A weak new peak located at 782.5 eV occurs in the XPS of Co 2*p* after 2500 CV cycles, demonstrating the existence of slight oxidation or OH^-absorption for Co atoms.

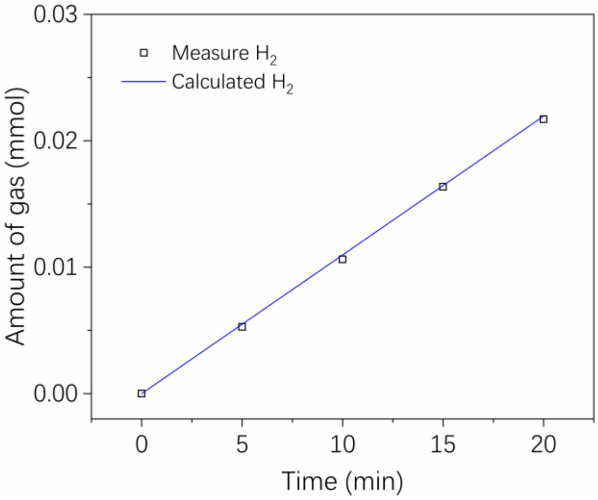

Supplementary Figure 40. The faradaic efficiency for HER.

The amount of hydrogen theoretically calculated and experimentally measured vs. time for n-Cu/BP in 1 M KOH for HER (50 mA cm^{-2} is passed over 20 minutes).

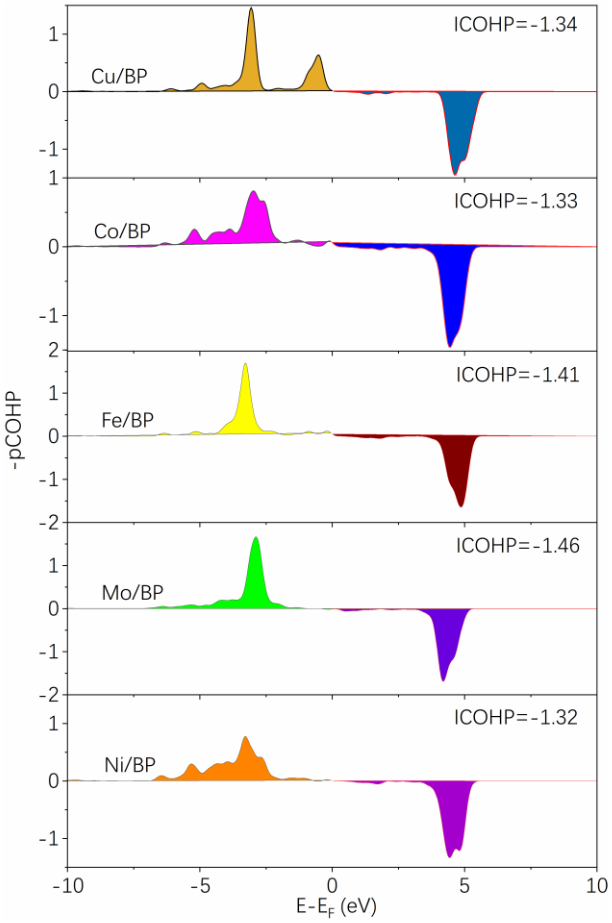

Supplementary Figure 41. The calculated projected crystal orbital Hamilton population.

Projected crystal orbital Hamilton population (COHP) for H*–substrate interactions in M/BP catalysts.

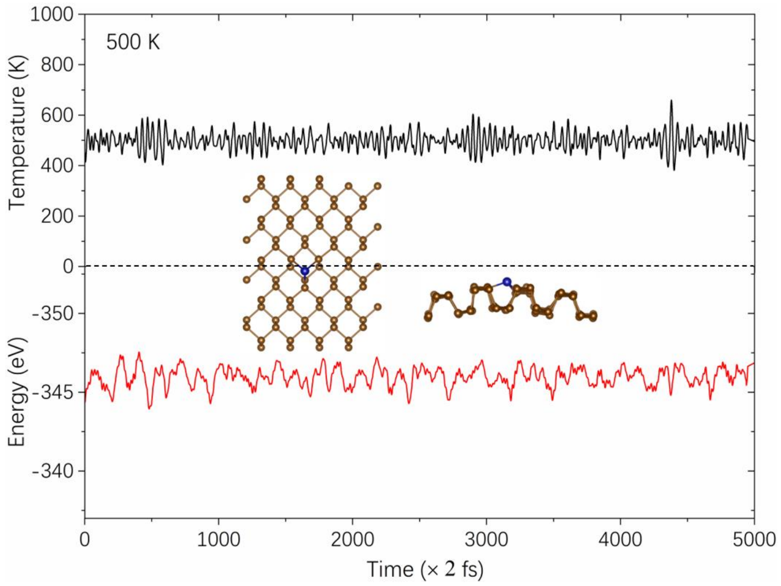

Supplementary Figure 42. The calculated Energy and temperature evolution versus the AIMD time for Cu/BP.

Insets show the top and side views of a snapshot of the atomic configuration. The simulation is run at 500 K for 10 ps with a time step of 2 fs.

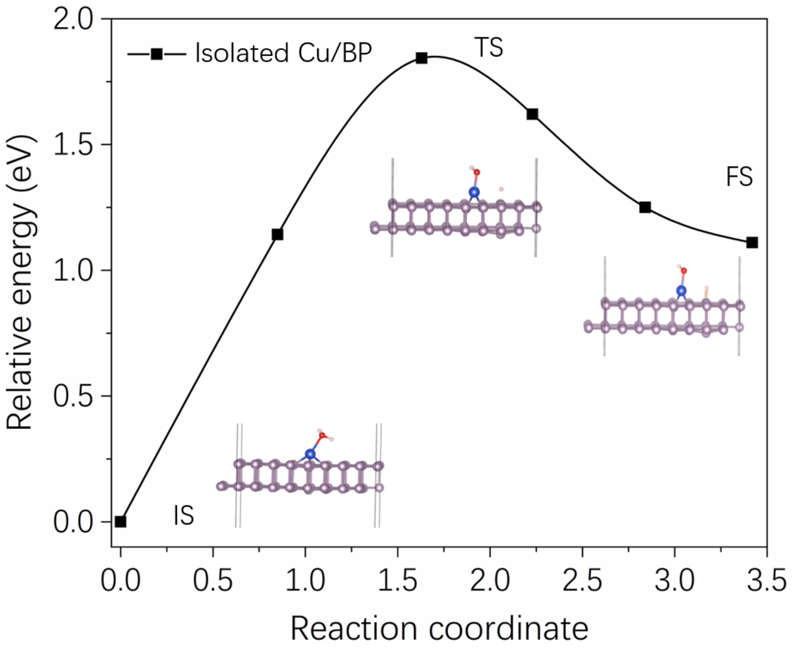

Supplementary Figure 43. Reaction energy diagrams of the different steps in alkaline HER on isolated Cu/BP.

The simplified surface structures of the various reaction species along the reaction pathway including H_2O adsorption state (IS), activated H_2O dissociation (TS), and H adsorption from final state (FS).

Supplementary Tables

Supplementary Table 1. The metal loading of n-Cu/BP and n-Co/BP catalysts under vis irradiation with H_2 auxiliary for 3h from ICP-OES results as compared with literature data.

Catalysts	Metal loading (wt%)	Ref.
n-Cu/BP	11.3	This work
n-Co/BP	5.2	This work
CuCo/BP	Cu 8.8, Co 3.7	This work
Cu SAs/UiO-66-NH_2	0.39	4
Cu-SA/SNC	4.5	5
PdCu/NC.	Cu 2.32, Pd 2.23	6
Cu/TiO_2	0.75	7
Ni-N_4/GHSs/Fe-N_4	Ni 1.9, Fe 3.0	8
Cu-SAs/N-C	0.95	9
Ni/GD	0.278	10
FeN_4/GN	4.0	11
Pt/MoS_2	7.5	12
Cu-ISAS@NaY	0.29	13
Fe-sMoS_2	9.34	14
Pd/TiO_2	1.5	15
Zn/CoN_x-C	Zn 0.33, Co 0.14	16
Co–N–C	1.63	17
Co–N–C@F127	6.2	18
Pt-ISAS	0.22	19
PtSA-NT-NF	1.6	20
CoSSPIL/CNT	4.0	21
SA-Fe-NHPC	1.25	22
Pt/HSC	5.0	23
Co-BP	4.0	24
Ni-C/N	2.2	25
WC_x-FeNi	Fe 1.37, Ni 1.73	26

Supplementary Table 2. The metal loading catalysts on BP support prepared under different vis irradiation time with H_2 auxiliary from ICP-OES results.

Vis irradiation	Cu/BP and Co/BP Species	Weight fraction (wt%)
0.5 h	Cu	1.52
	Co	0.71
1 h	Cu	3.93
	Co	1.82
5 h	Cu	15.8
	Co	7.6

Supplementary Table 3. Structural parameters extracted from the EXAFS fitting.

Sample	Shell	N^a	R (Å)^b	σ^2 (Å^2)^c	ΔE_0 (eV)^d	R factor
n-Cu/BP	Cu-P	3	2.34	0.00888	7.4	0.008
n-Co/BP	Co-P	3	2.16	0.00595	4.5	0.002

Supplementary Table 4. The metal loading Cu/BP and Co/BP catalysts on BP support prepared under different conditions from ICP-OES results.

Conditions	Species	Weight fraction (wt%)	Atomic fraction (at%)
Vis irradiation for 3h without H_2	Cu	1.39	0.68
	Co	0.65	0.35
H_2 without Vis irradiation	Cu	0.03	0.01
	Co	--	--

Supplementary Table 5. The metal single-atomic loading catalysts for CuCo/BP prepared from ICP-OES results.

Catalysts	Species	Weight fraction (wt%)
CuCo/BP	Cu	8.8
	Co	3.7

Supplementary Table 6. Comparison of HER activity data for various catalysts.

Catalysts	Overpotential (10 mA cm^{-2})	Tafel slope (mV dec^{-1})	References
n-Cu/BP	41	53.4	This work
n-Co/BP	141	131.6	This work
CuCo/BP	73	73.2	This work
Ni-MoS_2	98	60	[27]
Ru-MoS_2/CNT	50	62	[28]
Pt@PCM	139	73.6	[29]
sc-$Ni_2P^{\delta-}$-/NiHO	60	75	[30]
Co@C_2N	250	121	[31]
CoN_x/C	170	75	[32]
Cu NDs/Ni_3S_2 NTs-CFs	128	76.2	[33]
CoP/NCNHP	115	66	[34]
Ni@Ni_2P	150	213	[35]
W-SAC	85	53	[36]
Pt_1@Fe-N-C	108	/	[37]
Ni_{SA}-MoS_2/CC	95	75	[38]
Co_1/PCN	89	52	[39]
Pd/MoS_2	89	80	[40]
Ru_{SA}-N-S-$Ti_3C_2T_x$	76	90	[41]
MCM@MoS_2–Ni	161	81	[42]

Supplementary Table 7. Comparison of TOF of HER catalysts at overpotential 0.15 V in alkaline condition.

Catalysts	TOF (H_2 S^{-1})	References
n-Cu/BP	0.57	This work
CuCo/BP	0.17	This work
n-Co/BP	0.12	This work
Co_1/PCN	0.22	[39]
Ru@GnP	0.145 (0.1 V)	[43]
Co-Ni_3N	0.146 (0.2 V)	[44]
Ni-MoS2	0.32	[27]
$MoNi_4$/MoO_{3-x}	1.13 (0.1 V)	[45]
$Mo_1N_1C_2$	0.465	[46]
Ru/NG	0.35 (0.1 V)	[47]
Ni-C-N NSs	0.44 (0.1 V)	[48]
Co-NiS_2	0.55 (0.1 V)	[49]

Supplementary Table 8. The feeding amount of Cu and Co source of all the synthesized catalysts with different compounds and the corresponding actual atomic ratios of Cu:Co.

Samples	$Cu_0Co_{0.05}$ /BP	$Cu_{0.025}Co_{0.05}$ /BP	$Cu_{0.05}Co_{0.025}$ /BP	$Cu_{0.05}Co_{0.05}$ /BP	$Cu_{0.05}Co_{0.1}$ /BP	$Cu_{0.1}Co_{0.05}$ /BP	$Cu_{0.05}Co_0$ /BP
Cu (mmol)	0	0.025	0.05	0.05	0.05	0.1	0.05
Co (mmol)	0.05	0.05	0.025	0.05	0.1	0.05	0
Actual atomic ratios (Cu:Co)	--	1.02	3.82	2.19	1.21	4.34	--

Supplementary Table 9. Comparison of bond lengths and charge transfer (the "negative" represents the transfer of electrons from Cu to the support) for single-atom Cu-based catalysts.

Catalysts	Coordination	Bond length by FT-EXAFS (Å)	Charge transfer (|e|)	Reference
n-Cu/BP	Cu-P_3	1.93	-0.29	This work
Cu-N_4/C	Cu-N_4	1.42	-0.62	[50]
Cu-N_4/C-B		1.44	-0.89	
Cu-N_4/C-P		1.47	-0.59	
Cu_2@C_3N_4	N-Cu-N	1.62	-0.66	[51]
(Zn, Cu)-NC	Cu-C_2N	1.71(Cu-N)	-0.60	[52]
	Zn-N_4	1.44	-1.16	
Cu-SA/SNC	Cu-N_4	1.44	-1.14	[5]
PdCu/NC	Cu-N_2	1.47	-0.56	[6]
Cu-CDs	Cu-N_2	1.50	-0.87	[53]
	Cu-O_2	1.50		
CuNi-DSA/CNFs	CuN_4<br>NiN_4	1.50<br>1.52	-0.69<br>-0.66	[54]

Supplementary References

1. Li, L. K. et al. Black phosphorus field-effect transistors. *Nat Nanotechnol* **2014,** *9* (5), 372-377.
2. Kang, J. et al. Stable aqueous dispersions of optically and electronically active phosphorene. *P Natl Acad Sci USA* **2016,** *113* (42), 11688-11693.
3. Liu, F. L. et al. Direct Z-Scheme Hetero-phase Junction of Black/Red Phosphorus for Photocatalytic Water Splitting. *Angew Chem Int Edit* **2019,** *58* (34), 11791-11795.
4. Wang, G. et al. Photoinduction of Cu Single Atoms Decorated on UiO-66-NH2 for Enhanced Photocatalytic Reduction of CO2 to Liquid Fuels. *J Am Chem Soc* **2020,** *142* (45), 19339-19345.
5. Jiang, Z. L. et al. Atomic interface effect of a single atom copper catalyst for enhanced oxygen reduction reactions. *Energ Environ Sci* **2019,** *12* (12), 3508-3514.
6. Han, L. L. et al. Modulating Single-Atom Palladium Sites with Copper for Enhanced Ambient Ammonia Electrosynthesis. *Angew Chem Int Edit* **2021,** *60* (1), 345-350.
7. Lee, B. H.; Park, S.; Kim, M.; Sinha, A. K.; Lee, S. C.; Jung, E.; Chang, W. J.; Lee, K. S.; Kim, J. H.; Cho, S. P. et al. Reversible and cooperative photoactivation of single-atom Cu/TiO2 photocatalysts. *Nat Mater* **2019,** *18* (6), 620-+.
8. Chen, J. Y. et al. Dual Single-Atomic Ni-N(4)and Fe-N(4)Sites Constructing Janus Hollow Graphene for Selective Oxygen Electrocatalysis. *Adv Mater* **2020,** *32* (30).
9. Qu, Y. T. et al. Direct transformation of bulk copper into copper single sites via emitting and trapping of atoms. *Nat Catal* **2018,** *1* (10), 781-786.
10. Xue, Y. et al. Anchoring zero valence single atoms of nickel and iron on graphdiyne for hydrogen evolution. *Nat Commun* **2018,** *9* (1), 1460.
11. Deng, D. H. et al. A single iron site confined in a graphene matrix for the catalytic oxidation of benzene at room temperature. *Sci Adv* **2015,** *1* (11): e1500462.
12. Li, H. L. et al. Synergetic interaction between neighbouring platinum monomers in CO2 hydrogenation. *Nat Nanotechnol* **2018,** *13* (5), 411-+.
13. Li, J. Z. et al. Atomically dispersed manganese catalysts for oxygen reduction in proton-exchange membrane fuel cells. *Nat Catal* **2018,** *1* (12), 935-945.
14. Zheng, J. et al. High Loading of Transition Metal Single Atoms on Chalcogenide Catalysts. *J Am Chem Soc* **2021,** *143* (21), 7979-7990.
15. Liu, P. X. et al. Photochemical route for synthesizing atomically dispersed palladium catalysts. *Science* **2016,** *352* (6287), 797-801.
16. Lu, Z. et al. An Isolated Zinc-Cobalt Atomic Pair for Highly Active and Durable Oxygen Reduction. *Angew Chem Int Ed Engl* **2019,** *58* (9), 2622-2626.
17. Xie, X. et al. Performance enhancement and degradation mechanism identification of a single-atom Co–N–C catalyst for proton exchange membrane fuel cells. *Nat Catal* **2020,** *3* (12), 1044-1054.
18. He, Y. H. et al. Highly active atomically dispersed CoN4 fuel cell cathode catalysts derived from surfactant-assisted MOFs: carbon-shell confinement strategy. *Energ Environ Sci* **2019,** *12* (1), 250-260.
19. Liu, Y. W. et al. A General Strategy for Fabricating Isolated Single Metal Atomic Site Catalysts in Y Zeolite. *J Am Chem Soc* **2019,** *141* (23), 9305-9311.
20. Zhang, L. H. et al. Potential-Cycling Synthesis of Single Platinum Atoms for Efficient Hydrogen Evolution in Neutral Media. *Angew Chem Int Edit* **2017,** *56* (44), 13694-13698.

21. Ding, Y. X. et al. Cobalt-Bridged Ionic Liquid Polymer on a Carbon Nanotube for Enhanced Oxygen Evolution Reaction Activity. *Angew Chem Int Edit* **2018,** *57* (13), 3514-3518.
22. Chen, G. B. et al. Zinc-Mediated Template Synthesis of Fe-N-C Electrocatalysts with Densely Accessible Fe-N-x Active Sites for Efficient Oxygen Reduction. *Adv Mater* **2020,** *32* (8), 1907399.
23. Choi, C. H. et al. Tuning selectivity of electrochemical reactions by atomically dispersed platinum catalyst. *Nat Commun* **2016,** *7*, 10922.
24. Liu, D. N. et al. Direct Synthesis of Metal-Doped Phosphorene with Enhanced Electrocatalytic Hydrogen Evolution. *Small Methods* **2019,** *3* (7), 1900083.
25. Jin, X. et al. Electron Configuration Modulation of Nickel Single Atoms for Elevated Photocatalytic Hydrogen Evolution. *Angew Chem Int Ed Engl* **2020,** *59* (17), 6827-6831.
26. Li, S. et al. Oxygen-evolving catalytic atoms on metal carbides. *Nat Mater* **2021,** 1240-1247.
27. Zhang, J. et al. Engineering water dissociation sites in MoS2 nanosheets for accelerated electrocatalytic hydrogen production. *Energ Environ Sci* **2016,** *9* (9), 2789-2793.
28. Zhang, X. et al. Engineering MoS2 Basal Planes for Hydrogen Evolution via Synergistic Ruthenium Doping and Nanocarbon Hybridization. *Adv Sci* **2019,** *6* (10), 1900090.
29. Zhang, H. B. et al. Dynamic traction of lattice-confined platinum atoms into mesoporous carbon matrix for hydrogen evolution reaction. *Sci Adv* **2018,** *4* (1), eaao6657.
30. You, B. et al. Negative Charging of Transition-Metal Phosphides via Strong Electronic Coupling for Destabilization of Alkaline Water. *Angew Chem Int Edit* **2019,** *58* (34), 11796-11800.
31. Mahmood, J. et al. An efficient and pH-universal ruthenium-based catalyst for the hydrogen evolution reaction. *Nat Nanotechnol* **2017,** *12* (5), 441-446.
32. Liang, H. W. et al. Molecular metal-N-x centres in porous carbon for electrocatalytic hydrogen evolution. *Nat Commun* **2015,** *6*, 7992.
33. Feng, J. X. et al. Efficient Hydrogen Evolution on Cu Nanodots-Decorated Ni3S2 Nanotubes by Optimizing Atomic Hydrogen Adsorption and Desorption. *J. Am. Chem. Soc.* **2018,** *140* (2), 610-617.
34. Pan, Y. et al. Core-Shell ZIF-8@ZIF-67-Derived CoP Nanoparticle-Embedded N-Doped Carbon Nanotube Hollow Polyhedron for Efficient Overall Water Splitting. *J. Am. Chem. Soc.* **2018,** *140* (7), 2610-2618.
35. Liu, Y. et al. Ru Modulation Effects in the Synthesis of Unique Rod-like Ni@Ni2P-Ru Heterostructures and Their Remarkable Electrocatalytic Hydrogen Evolution Performance. *J. Am. Chem. Soc.* **2018,** *140* (8), 2731-2734.
36. Chen, W. X. et al. Single Tungsten Atoms Supported on MOF-Derived N-Doped Carbon for Robust Electrochemical Hydrogen Evolution. *Adv. Mater.* **2018,** *30* (30), 1800396.
37. Zeng, X. J. et al. Single-Atom to Single-Atom Grafting of Pt-1 onto Fe-N-4 Center: Pt-1@Fe-N-C Multifunctional Electrocatalyst with Significantly Enhanced Properties. *Adv Energy Mater* **2018,** *8* (1), 1701345.
38. Wang, Q. et al. Design of active nickel single-atom decorated MoS2 as a pH-universal catalyst for hydrogen evolution reaction. *Nano Energy* **2018,** *53*, 458-467.
39. Cao, L. L. et al. Identification of single-atom active sites in carbon-based cobalt catalysts during electrocatalytic hydrogen evolution. *Nat Catal* **2019,** *2* (2), 134-141.
40. Luo, Z. Y. et al. Chemically activating MoS2 via spontaneous atomic palladium interfacial doping towards efficient hydrogen evolution. *Nat Commun* **2018,** *9,* 2120.
41. Ramalingam, V. et al. Heteroatom-Mediated Interactions between Ruthenium Single Atoms

and an MXene Support for Efficient Hydrogen Evolution. *Adv. Mater.* **2019,** *31* (48), 1903841.
42. Zhang, H. B. et al. Surface Modulation of Hierarchical MoS2 Nanosheets by Ni Single Atoms for Enhanced Electrocatalytic Hydrogen Evolution. *Adv. Funct. Mater.* **2018,** *28* (51), 1807086.
43. Li, F. et al. Mechanochemically Assisted Synthesis of a Ru Catalyst for Hydrogen Evolution with Performance Superior to Pt in Both Acidic and Alkaline Media. *Adv. Mater.* **2018,** *30* (44), 1803676.
44. Zheng, X. Q. et al. Role of non-metallic atoms in enhancing the catalytic activity of nickel-based compounds for hydrogen evolution reaction. *Chem Sci* **2018,** *9* (7), 1822-1830.
45. Chen, Y. Y. et al. Self-Templated Fabrication of MoNi4/MoO3-X Nanorod Arrays with Dual Active Components for Highly Efficient Hydrogen Evolution. *Adv. Mater.* **2017,** *29* (39), 1703311.
46. Chen, W. X. et al. Rational Design of Single Molybdenum Atoms Anchored on N-Doped Carbon for Effective Hydrogen Evolution Reaction. *Angew Chem Int Edit* **2017,** *56* (50), 16086-16090.
47. Ye, R. Q. et al. High Performance Electrocatalytic Reaction of Hydrogen and Oxygen on Ruthenium Nanoclusters. *Acs Appl Mater Inter* **2017,** *9* (4), 3785-3791.
48. Yin, J. et al. Ni-C-N Nanosheets as Catalyst for Hydrogen Evolution Reaction. *J. Am. Chem. Soc.* **2016,** *138* (44), 14546-14549.
49. Yin, J. et al. Atomic Arrangement in Metal-Doped NiS2 Boosts the Hydrogen Evolution Reaction in Alkaline Media. *Angew Chem Int Edit* **2019,** *58* (51), 18676-18682.
50. Zhou, X. et al. Identification of Fenton-like active Cu sites by heteroatom modulation of electronic density. *P Natl Acad Sci USA* **2022,** *119* (8).
51. Xie, P. F. et al. Oxo dicopper anchored on carbon nitride for selective oxidation of methane. *Nat Commun* **2022,** *13* (1), 1375.
52. Deng, D. J. et al. Non-Covalent Interaction of Atomically Dispersed Cu and Zn Pair Sites for Efficient Oxygen Reduction Reaction. *Adv. Funct. Mater.* **2022**, 220371.
53. Cai, Y. M. et al. Insights on forming N,O-coordinated Cu single-atom catalysts for electrochemical reduction CO2 to methane. *Nat Commun* **2021,** *12* (1), 586.
54. Hao, J. et al. Interatomic Electronegativity Offset Dictates Selectivity When Catalyzing the CO 2 Reduction Reaction. *Adv Energy Mater* **2022**, 2200579.
